# Hypophysectomy abolishes rhythms in rat thyroid hormones but not in the thyroid clock

**DOI:** 10.1530/JOE-17-0111

**Published:** 2017-03-27

**Authors:** J Fahrenkrug, B Georg, J Hannibal, H L Jørgensen

**Affiliations:** Department of Clinical BiochemistryBispebjerg and Frederiksberg Hospital, Faculty of Health Sciences, University of Copenhagen, Copenhagen, Denmark

**Keywords:** thyroid hormones, circadian rhythms, clock genes, HPT axis, pituitary

## Abstract

The endocrine body rhythms including the hypothalamic–pituitary–thyroid axis seem to be regulated by the circadian timing system, and daily rhythmicity of circulating thyroid-stimulating hormone (TSH) is well established. The circadian rhythms are generated by endogenous clocks in the central brain oscillator located in the hypothalamic suprachiasmatic nucleus (SCN) as well as multiple peripheral clocks, but information on the existence and function of a thyroid clock is limited. The molecular machinery in all clock cells is composed of a number of clock genes and their gene products are connected by autoregulatory feedback loops. Here, we provide evidence for a thyroid clock in the rat by demonstrating 24-h antiphase oscillations for the mRNA of the canonical clock genes *Per1* and *Bmal1*, which was unaffected by hypophysectomy. By immunostaining, we supported the existence of a core oscillator in the individual thyroid cells by demonstrating a daily cytoplasmatic–nuclear shuttling of PER1 protein. In normal rats, we found a significant daily rhythmicity in the circulating thyroid hormones preceded by a peak in TSH. In hypophysectomised rats, although the thyroid clock was not affected, the oscillations in circulating thyroid hormones were abolished and the levels were markedly lowered. No daily oscillations in the expression of TSH receptor mRNA were observed in neither control rats nor hypophysectomised rats. Our findings indicate that the daily rhythm of thyroid hormone secretion is governed by SCN signalling via the rhythmic TSH secretion rather than by the local thyroid clock, which was still ticking after hypophysectomy.

## Introduction

There is growing evidence that endocrine body rhythms including the hypothalamic–pituitary–thyroid (HPT) axis are regulated by the circadian timing system, which consists of the master pacemaker located in the hypothalamic suprachiasmatic nucleus (SCN) as well as multiple peripheral clocks (Prasai *et al*. 2011, Gamble *et al*. 2014, Tsang *et al*. 2014). The SCN clock is adjusted daily by photic cues transmitted from the retina via a monosynaptic nervous pathway, the retinohypothalamic tract (Golombek & Rosenstein 2010). In turn, the SCN determines the phase of the peripheral organs through a variety of signalling pathways including blood-borne signals and the peripheral nervous system (Dibner *et al*. 2010). In all clock cells, the molecular machinery is composed of the same clock genes and their protein products connected by autoregulatory feedback loops. The major loop comprises the PAS domain helix-loop-helix transcriptional activators BMAL1 and CLOCK forming heterodimers that activate the transcription of E-box-containing cryptochromes (*Cry1* and *Cry2*) and periods (*Per1* and *Per2*) as well as clock-controlled output genes (Reppert & Weaver 2001, Lowrey & Takahashi 2004, Okamura 2004). After synthesis in the cytoplasm, CRY and PER proteins translocate into the nucleus and form inhibitory complexes feeding negatively back on their own transcription.

A daily rhythmicity of circulating thyroid-stimulating hormone (TSH) peaking at daytime in rats is well established (Rookh *et al*. 1979, Jordan *et al*. 1980, Ottenweller & Hedge 1982, Wong *et al*. 1983, Kalsbeek *et al*. 2000, Guo *et al*. 2015). Nerve fibres originating in the SCN innervating thyroid-releasing hormone (TRH) neurons in the paraventricular nucleus are the anatomical basis for the daily rhythms in TRH content and secretion, which subsequently regulate the rhythmic secretion of TSH from the pituitary (Kalsbeek & Fliers 2013). TSH stimulates the thyroid gland to synthesise and secrete thyroid hormones. Studies in rats on the existence of a daily oscillating pattern of thyroid hormones in the circulation have, however, not been quite unequivocal, although most favour a diurnal peak of thyroxin (T4) and triiodothyronine (T3) during the light period (Jordan *et al*. 1980, Ottenweller & Hedge 1982, Wong *et al*. 1983, Kalsbeek *et al*. 2000, Guo *et al*. 2015).

There is now evidence that peripheral clocks in some endocrine cells can regulate the rhythmic transcription of genes controlling hormone secretion (Perelis *et al*. 2015). Information on the existence of thyroid clocks and their cellular localisation is limited and primarily based on circadian oscillations of clock genes in cultured human thyrocytes and luminescence cycles produced by a *Bmal1*-luciferase reporter (Mannic *et al*. 2013). Furthermore, the possible functional role of a thyroid clock and its dependence on input from the pituitary is completely unknown.

In the present study, we provide evidence for the existence of a pituitary-independent thyroid clock in the rat by demonstrating 24-h antiphase oscillations for the transcripts of the canonical clock genes *Per1* and *Bmal1* and by visualising a daily cytoplasmatic–nuclear shuttling of PER1 protein in thyroid cells. Furthermore, we found that although the thyroid clock was not affected, the daily rhythmicity of circulating thyroid hormone T4, free T4 and T3 was eliminated by hypophysectomy indicating that the daily pattern in thyroid hormones is depending on SCN signalling via the pituitary gland rather than the local thyroid clock.

## Materials and methods

### Animals

Adult Wistar rats (Taconic Breeding Centre, Ll. Skensved, Denmark, Harlan, Netherlands or Charles River Laboratories Research Models and Services, Sulzfeld, Germany) of both sexes weighing 150–200 g were housed under standard laboratory conditions with *ad libitum* access to food and water. The animals were treated according to the Ethical Principles of Laboratory Animal Care Law on Animal experiments in Denmark, publication 1306, November 23, 2007. Three experimental groups were included in the study:

1. Rats entrained for at least 14 days to a 12:12-h light/darkness cycle (Zeitgeber time ZT0 = lights on and ZT12 = lights off) were killed by decapitation at each of the following time points: ZT0, ZT2, ZT6, ZT10, ZT12, ZT14, ZT16, ZT18 and ZT20.
2. Rats entrained to a 12-h:12-h light/dark cycle for at least 14 days after which the light was turned off. During the second cycle after a transfer into continuous darkness, animals were killed at time points corresponding to experimental group 1.
3. Rats were hypophysectomised by the parapharyngeal method one week prior to shipment from the vendor. We ensured the completeness of the hypophysectomy after killing by inspection with a dissecting microscope and, furthermore, serum TSH was unmeasurable. The drinking water was supplemented with 5% glucose and after 3 weeks of entrainment to a 12-h:12-h light/darkness cycle, the animals were killed at the same time points as for experimental groups 1 and 2.

The thyroid glands were dissected from ice-cold tissue blocks rapidly removed and placed on dry ice after decapitation of the animals. The glands from animals in all experimental groups were used for quantification of *Per1* and *Bmal1* mRNA by qPCR. In addition, thyrotropin receptor (TSH-R) mRNA in glands from experimental groups 1 and 2 was quantified. Thyroid glands for immunocytochemistry were obtained from control rats during a 12-h:12-h light/darkness cycle. Three animals were perfusion fixed with Stefanini’s fixative at each time point corresponding to the experimental groups.

### RNA extraction, cDNA synthesis and qPCR

Total RNA from each thyroid gland was prepared by the guanidinium thiocyanate–phenol–chloroform extraction method (Chomczynski & Sacchi 1987). cDNA was prepared using High-Capacity cDNA Archive Kit (Thermo Fisher Scientific) using 1 μg total RNA in a total reaction volume of 100 μL, and qPCR was performed using an ABI7000 or StepOnePlus instrument using TaqMan-based chemistry (Thermo Fisher Scientific). The assays and standard curves for *Per1*, *Bmal1* and the β2-microglobulin (*B2m*) internal control have all been described previously (Fahrenkrug *et al*. 2006,^ 2008^). TSH-R mRNA was determined using TaqMan Gene Expression Assay Rn00563612_m1 (Thermo Fisher Scientific). The standard curve for this assay was made as previously described for Per1 and Bmal1 (Fahrenkrug *et al.* 2006) using thyroid RNA purified from tissues samples collected at several time points both during day and night. Reactions were run in 20 μL containing cDNA from 20 ng total RNA and using TaqMan Universal PCR Master Mix containing AmpErase7UNG (Thermo Fisher Scientific). *Per1*, *Bmal1* or TSH-R and the *B2m* assays were run in separate wells on the same plate, and all samples, standards and the non-template negative controls were made in duplicates. The ABI prism 7000 SDS or StepOne software program (Thermo Fisher Scientific) was used to calculate the concentrations (in arbitrary units) of *Per1*, *Bmal1* or TSH-R and *B2m* obtained from the same run leading to a normalised target gene mRNA quantity.

### Immunohistochemistry

Thyroid glands from rats transcardially perfused with Stefanini’s fixative were cut in a cryostat as 12 μm thick sections. The fixed sections were processed for PER1 immunohistochemistry as previously described using PER1 antiserum raised and characterised in our own laboratory (Fahrenkrug *et al*. 2006) and counterstained using 4′,6′-diamidino-2-phenylindole (DAPI). Fluorescence images were obtained using an iMIC confocal microscope (TILL Photonics, FEI, Germany) and appropriate filter settings for detection of Alexa 488 and DAPI. Images were edited for contrast and brightness by Adobe Photoshop (Adobe Systems) and combined into plates using Adobe Illustrator (Adobe Systems).

### Hormone assays

Blood samples were obtained at ZT0, ZT2, ZT4, ZT6, ZT8, ZT10, ZT12, ZT16, ZT20 and ZT24 from control rats and at ZT0, ZT2, ZT6, ZT10, ZT12, ZT16 and ZT20 from hypophysectomised rats during a 12-h:12-h light/darkness cycle. The control rats were followed closely to more precisely determine the peak time of hormone concentrations and their time relation. The blood samples were allowed to clot at room temperature for 30 min prior to centrifugation at 1000 ***g***. The supernatant was stored at −20°C until assayed. Serum concentrations of TSH were measured by a solid-phase enzyme immunometric assay for use in rats (Demeditec Diagnostics, Kiel, Germany). The interassay coefficient of variation was 8.3% and the minimum detectible concentration was 0.1 ng/mL. T4, free T4 and T3 were determined on the Cobas 8000 (Roche Diagnostics) by electro-chemiluminescence immunoassays having interassay coefficient of variations of 4.6, 2.1 and 6.0% in the normal range and 7.5, 5.0 and 7.5% in the low range, respectively.

### Statistical analysis

Values were presented as the means ± standard error of mean (s.e.m.). Diurnal and/or circadian changes in clock gene mRNAs and hormone concentrations were analysed using the methods for cosinor rhythmometry as described by Nelson and coworkers (Nelson *et al*. 1979). The data were thus fitted to a combined cosine and sine function: Per = M + k1COS(2πt/24) + k2SIN(2πt/24). Substituting COS(2πt/24) = C and SIN(2πt/24) = Z gives the expression: Per = M + k1C + k2Z. The model fit was then tested using the general linear model procedure in the SAS statistical software package (SAS/STAT User’s Guide, Cary, NC: SAS Institute, 1994). *P* < 0.05 was considered statistically significant.

## Results

**Figure 1:**
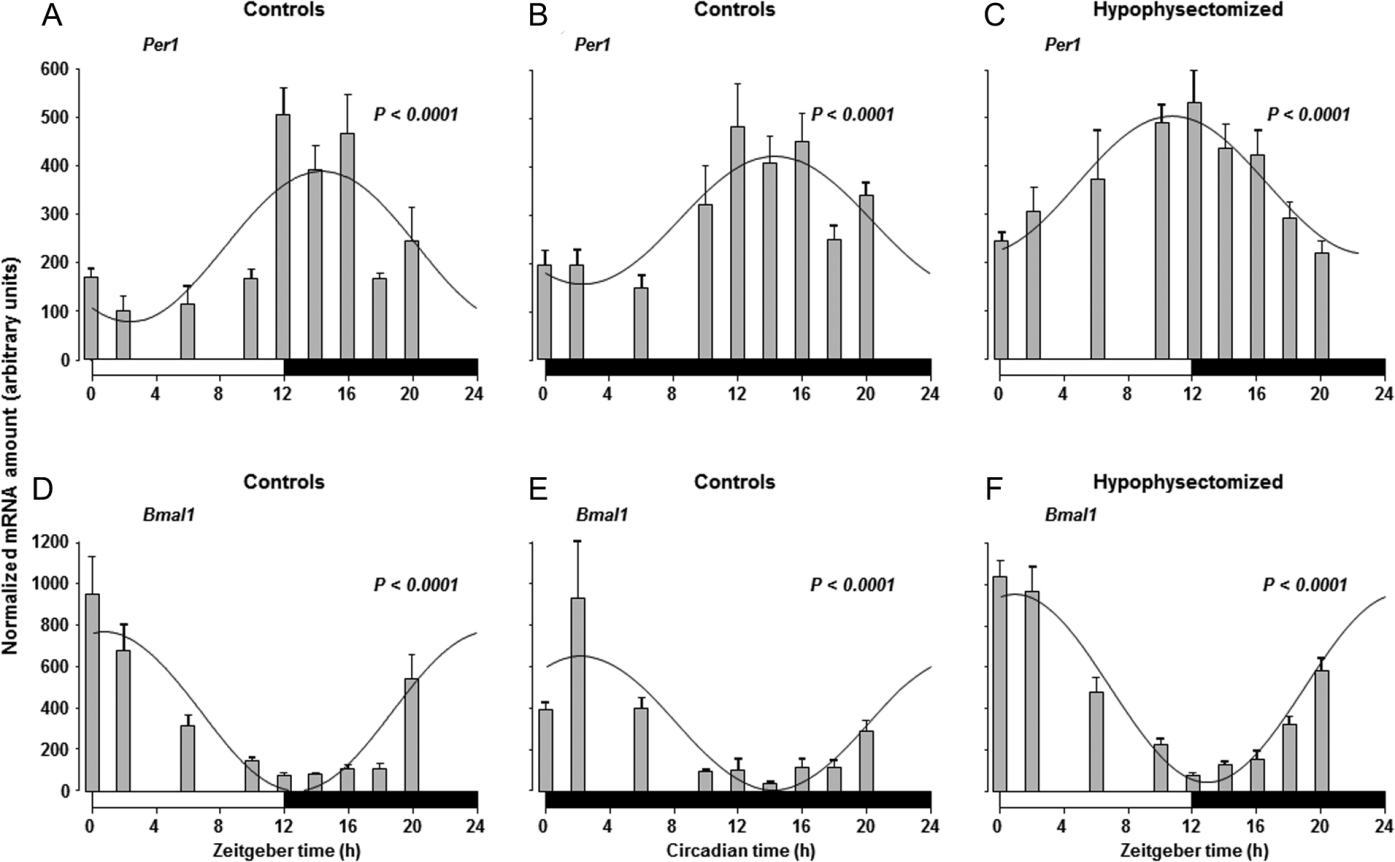
Rhythmic changes in the expression of the clock genes *Per1* (A, B, C) and *Bmal1* (D, E, F) in the thyroid gland from control (A and B, D and E) and hypophysectomised rats (C and F) during a 12-h:12-h light/darkness cycle (A and C; D and F) and during continuous darkness (B and E). *Per1* and *Bmal1* mRNA were quantified using real-time reverse transcription-polymerase chain reaction at each time point values are given as mean ± s.e.m. (*n* = 5). mRNA levels for the two clock genes were rhythmic and changed significantly as a function of a 24-h cycle. Fitted curves have been drawn. The white and black bars at the bottom of the graphs represent the periods of light and darkness, respectively.

The canonical circadian clock genes *Per1* and *Bmal1* were expressed in the rat thyroid gland and quantification of their mRNAs during a 12-h:12-h daily cycle disclosed a statistically significant cyclic oscillation as a function of a 24-h cycle (Fig. 1A and D). The *Per1* mRNA level was low at ZT0 and peaked around ZT12, whereas *Bmal1* as expected was in antiphase with *Per1* mRNA showing nadir and peak values around ZT12 and ZT0, respectively. After transfer into constant darkness the rhythmic expression of *Per1* and *Bmal1* continued with a pattern identical to that observed during the light/dark cycle (Fig. 1B and E).

**Figure 2:**
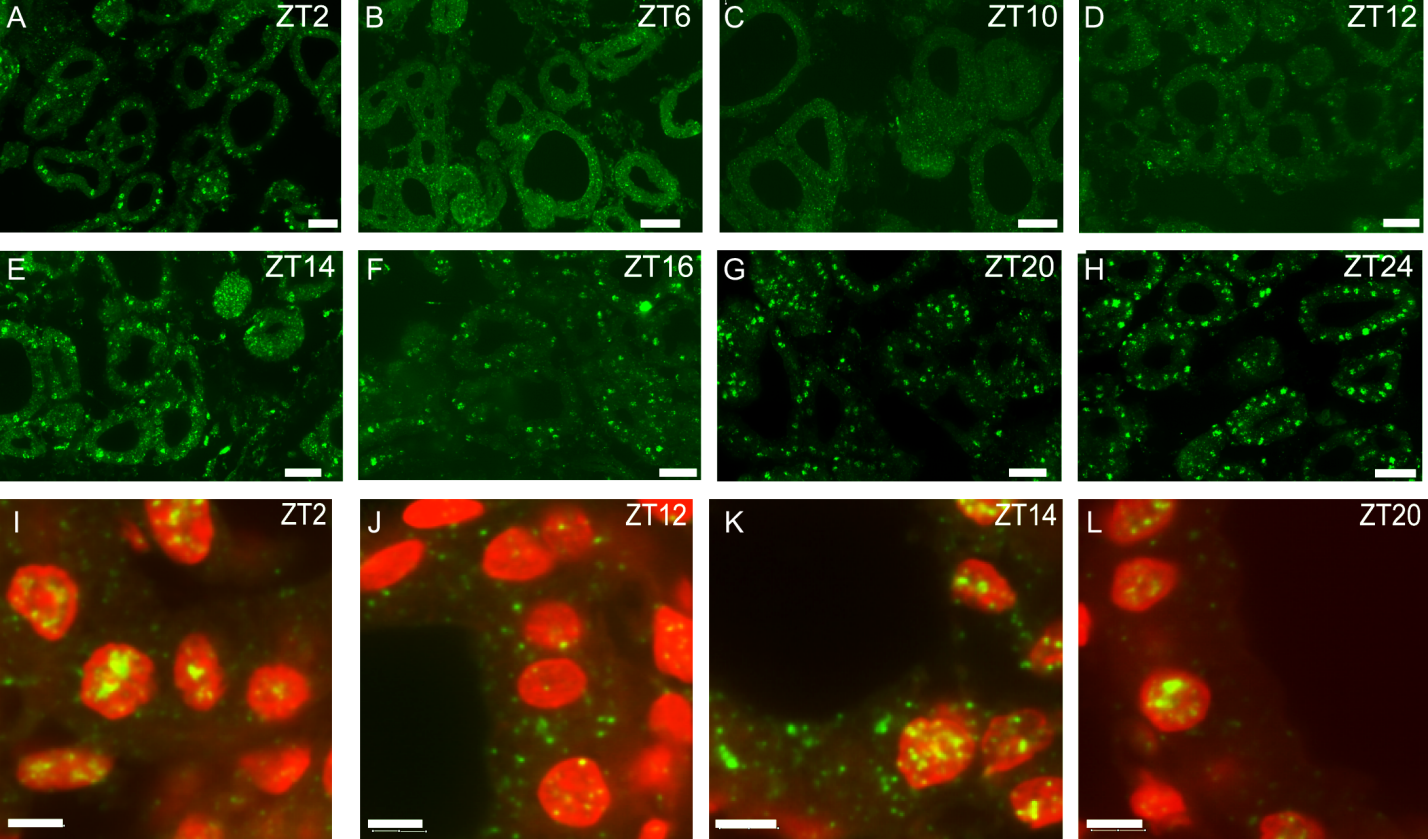
Confocal images of immunofluorescent staining of rat thyroid glands for PER1 (green) taken at representative time points during a 12-h:12-h light/darkness cycle (A, B, C, D, E, F, G and H). Note the changes in staining intensity and intracellular localization of Per1 illustrating the cytoplasmatic–nuclear shuttling over time. (I, J, K and L) represent high-power confocal micrographs of PER1 immunostaining (green) in thyroid follicular cells and DAPI nuclear staining (red) at selected time points. Note the intracellular changes in PER1 protein during a daily cycle where PER1 immunostaining was detected in the cytoplasm at ZT12 and in both the cytoplasm and nucleus at ZT14 followed by strong nuclear PER1 immunostaining at ZT20 and ZT2. Scale bars: A, B, C, D, E, F, G and H: 25 µm; I, J, K and L: 5 µm.

PER1 protein was expressed in the rat thyroid follicle cells as examined by immunohistochemistry and displayed marked changes in level and localisation across the daily cycle (Fig. 2). At Z6 to ZT12 immunostaining for PER1 was weak and primarily located in the cytoplasm. Subsequently, PER1 was transferred to the nuclei of the cells and at ZT14 PER1 was present in both the cytoplasm and the nuclei followed by intense PER1 immunostaining in the nuclei at ZT20 to ZT2.

**Figure 3:**
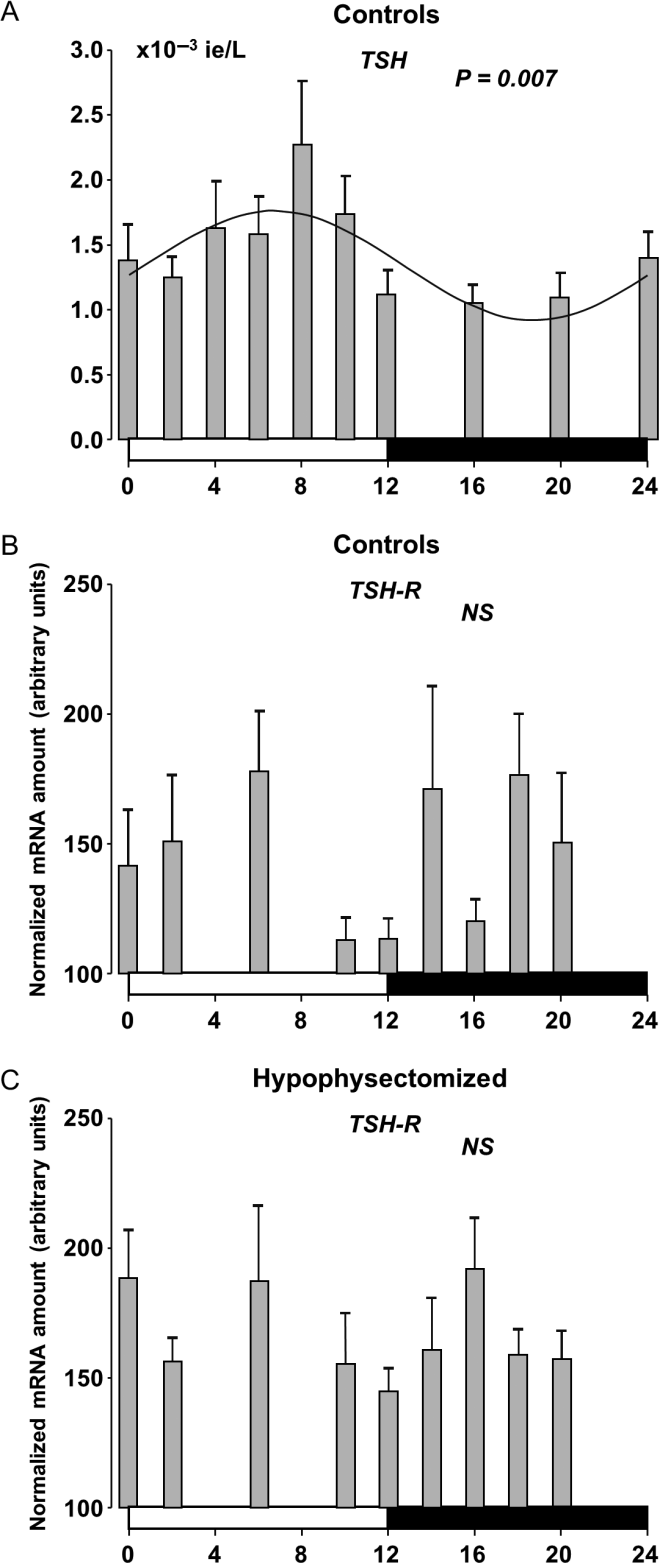
Twenty-four-hour rhythms in serum TSH concentration in control rats (A), and expression in thyrotropin receptor (TSH-R) mRNA of control rats (B) and in hypophysectomised rats (C) during a 12-h:12-h light/darkness cycle. At each time point, values are given as mean ± s.e.m. (*n* = 5–15). A fitted curve has been drawn in A. The white and black bars at the bottom of the graphs represent the period of light and darkness, respectively. NS, not significant.

**Figure 4:**
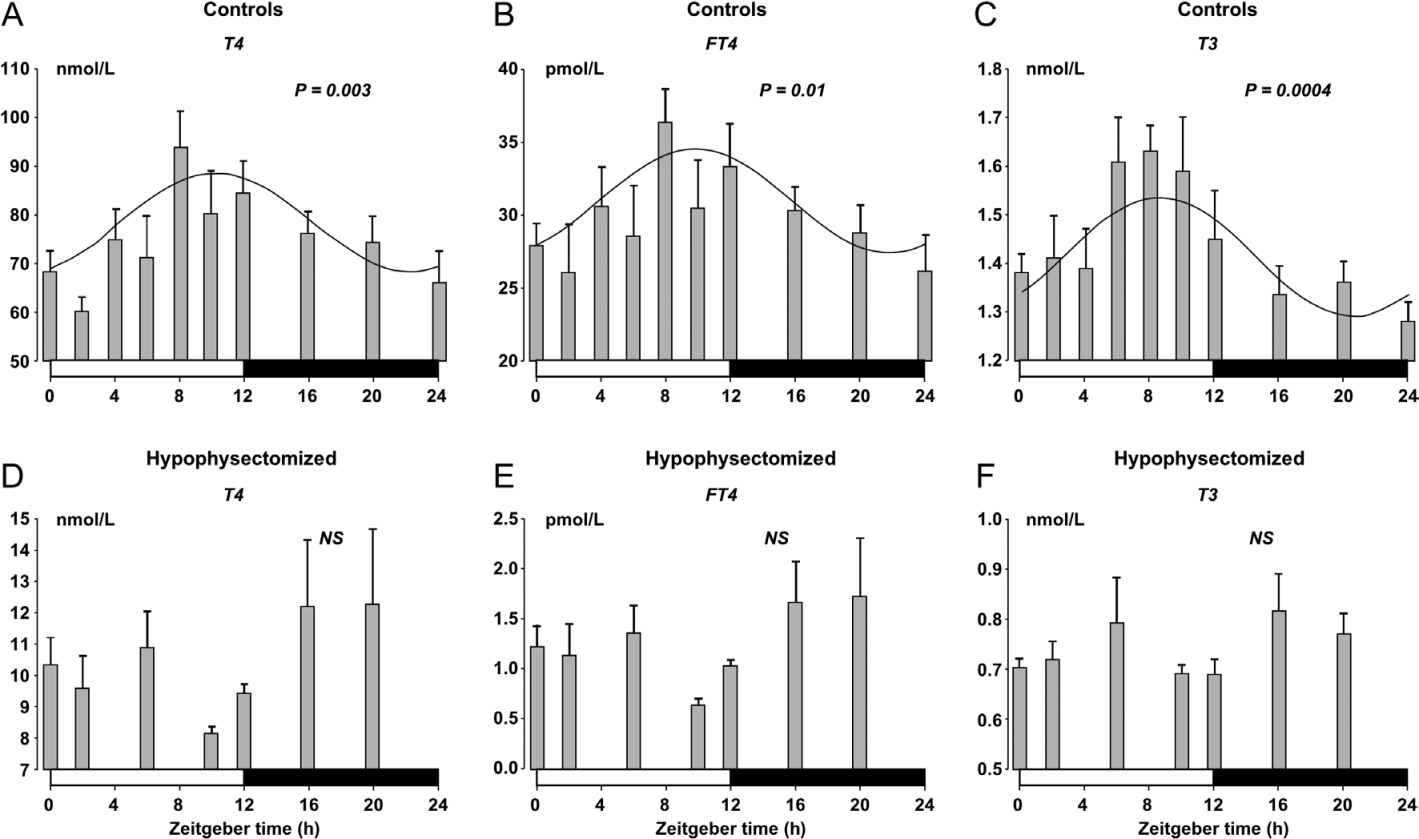
Twenty-four-hour rhythms in the concentration of T4, free T4 and T3 in serum of control (A, B, C) and hypophysectomised rats (D, E, F). Blood was sampled at the depicted time points under 12-h:12-h light/darkness conditions. At each time point, values are given as mean ± s.e.m. (*n* = 4–12). Fitted curves have been drawn in A, B and C. The white and black bars at the bottom of the graphs represent the periods of light and darkness, respectively. NS: not significant.

The daily profiles of circulating TSH, T4, free T4 and T3 and the chronobiological parameters of control rats are shown in Figs 3A, 4A, B, C and Table 1. During the light/darkness cycle, a marked increase in TSH levels was observed at the middle of the light phase with a peak concentration at ZT6 after which it gradually decreased (Fig. 3A). Similar and somewhat phase-delayed rhythmic variations in the levels T4, free T4 and T3 were apparent (Fig. 4A, B and C). All hormone profiles changed significantly as a function of a 24-h cycle, whereas no daily rhythm in the expression of TSH-R mRNA was observed (Fig. 3B).

**Table 1:** The chronobiological parameters of TSH and thyroid hormones in control rats.

	**TSH**	**T4**	**FT4**	**T3**
*T*_max_ (ZT)	6:41	10:10	9:50	8:37
*T*_min_ (ZT)	18:41	22:10	21:50	20:37
Amplitude*	0.8	20	7.1	0.2
Mesor/mean	1.4	77	30	1.4
Significance	0.007	0.003	0.01	<0.001

* The amplitude was calculated as the value of the fitted combined cosine and sine function at *t*_max_ minus its value at *t*_min_.

After hypophysectomy, *Per1* and *Bmal1* mRNAs in the thyroid gland displayed an unaltered 24-h rhythmic pattern of oscillation compared to intact rats, and the phases did not differ in the two groups of animals (Fig. 1C and F). In the hypophysectomised rats as in controls, no rhythmic pattern in TSH-R mRNA was apparent (Fig. 3C).

In hypophysectomised rats, TSH concentration in serum was undetectable confirming the ablation of the anterior pituitary. Hypophysectomy resulted in a marked lowering of the levels of circulating thyroid hormones compared to control rats, and based on the mean concentrations, it amounted to 7.7-fold for T4, 23-fold for free T4 and two-fold for T3, respectively (Fig. 4D, E and F). Furthermore, daily circadian rhythms in the thyroid hormones were eliminated after hypophysectomy (Fig. 4D, E and F).

Due to the observed variation in T4, free T4 and T3 after hypophysectomy, we tested for a possible 12-h rhythmicity. This was not present for T4 or for free T4. However, there was a slightly significant fit for T3 to a 12-h cycle (*P* = 0.04). The same test was performed for TSH-R mRNA expression which in neither the hypophysectomised rats nor the controls exhibited a significant 12-h rhythmicity.

## Discussion

In the present study, we demonstrated that the canonical circadian clock genes *Per1* and *Bmal1* displayed a 24-h rhythmic expression in the rat thyroid gland, which was unaltered after removal of the pituitary gland. The antiphase expression pattern of the two clock genes and the persistence of their rhythmic changes during constant darkness are consistent with the existence of a circadian clock in the rat thyroid gland.

Examination of the expression of PER1 protein by immunohistochemistry showed that PER1 occurred in the follicular epithelial cells where it displayed a characteristic time course of changes in the level and intracellular localisation. Thus, from a just detectable level, the PER1 protein accumulated within the cytoplasm after which the PER1 was transferred to the nuclei. The rise and fall of the PER 1 protein followed the cycle of mRNA expression with a lag of several hours. The nuclear accumulation of PER1 protein coincided with declining levels for PER1 mRNA, which is consistent with the delayed feedback model of the oscillator. These findings are in accordance with our previous observations in the rat ovaries (Fahrenkrug *et al*. 2006).

As the expression and rhythmicity of the local clock in the thyroid gland was unaffected by hypophysectomy, it seems not to be directly related to or regulated by the HPT axis. However, our demonstration of rhythmic oscillations of major clock components in the thyroid hormone-producing cells raises the question whether this local clock plays a role in thyroid hormone synthesis and/or secretion.

By measuring the circulating concentration of TSH, T4, free T4 and T3, we found that all hormones displayed rhythmic fluctuations that changed significantly over 24 h. The daily oscillation of TSH peaking at midday in the rats resting period was faithfully followed by peaks in the thyroid hormones 2–4 h later, which could indicate that the diurnal peaks of thyroid hormones are related to TSH-induced changes in thyroid hormone production and/or secretion. The significant changes in free T4 rule out that the daily rhythmicity of the total thyroid hormones could be due to variations in thyroid hormone-binding protein concentration in the circulation. The observed daily rhythmicity of TSH in rats accords well with previous studies (Fukuda *et al*. 1975, Rookh *et al*. 1979, Jordan *et al*. 1980, Ottenweller & Hedge 1982, Kalsbeek *et al*. 2000, Guo *et al*. 2015). The literature on the daily rhythm of total T4 and/or T3 has, however, been more equivocal. Some studies fail to find diurnal variations (Fukuda *et al*. 1975, Rookh *et al*. 1979), whereas most and later studies report a significant daily rhythm in T4 and/or T3 (Jordan *et al*. 1980, Ottenweller & Hedge 1982, Wong *et al*. 1983, Kalsbeek *et al*. 2000, Guo *et al*. 2015). In the present study, we found highly significant parallel diurnal changes in T4, free T4 and T3, which for at least T4 most likely represent variation in thyroid secretion. The peak concentrations of TSH and thyroid hormones occurred in the rat during the light period, which aligns with their resting period and accordingly the phasing of the rhythm is reversed in humans (Russell *et al*. 2008).

In addition, we demonstrated that the rhythmicity of circulating thyroid hormones was eliminated by hypophysectomy and that their levels were markedly lowered. This indicates that the daily pattern of thyroid hormone secretion is governed by SCN signalling via the rhythmic TSH secretion from the pituitary and not by the local thyroid clock, which was still ticking after hypophysectomy. Furthermore, it has previously been shown that elimination of the master biological clock by thermal ablation of SCN abolished the diurnal peak in circulating TSH and thyroid hormones (Kalsbeek *et al*. 2000).

Evidence for the existence of the polysynaptic SCN–thyroid pathway has been provided using retrograde transneuronal viral tracer (Kalsbeek *et al*. 2000). Although hypophysectomy abolished the daily rhythm of the thyroid hormones, a role for this polysynaptic SCN–thyroid pathway in rhythmic thyroid hormone secretion or adjustment of diurnal sensitivity of the TSH receptor and/or post-receptor events is still possible.

It has previously been shown that the clock in the adrenal gland regulates the ACTH sensitivity, which drives daily oscillation in glucocorticoid secretion (Dickmeis 2009). Thus, it is possible that the local thyroid clock could be responsible for a time of day-dependent oscillation in TSH sensitivity. Consequently, we examined whether there was a diurnal variation in the TSH receptor mRNA expression in control rats with oscillations in both TSH levels and clock genes and in hypophysectomised rats with the absence of TSH, but with intact thyroid clock rhythmicity. We found that the expression level of TSH-R mRNA was constant throughout the daily cycle in both control and hypophysectomised rats, which makes it unlikely to be controlled by the thyroid clock. Furthermore, the TSH-R level seems not to be influenced by the diurnal changes in TSH concentration. There is evidence from ligand-binding studies that the TSH-R can be regulated by TSH as well as the thyroid hormones, but not within the physiological range which accords well with our finding on the TSH-R mRNA (Witte *et al*. 1980, Witte & McKenzie 1981, Denereaz & Lemarchand-Beraud 1995).

In conclusion, we have provided evidence for the presence of a molecular clock in the thyroid follicular cells of the rat, which was independent of input from the pituitary. Furthermore, our findings indicate that the daily rhythms of thyroid hormone secretion is governed by SCN signalling via the rhythmic TSH secretion from the pituitary rather than by the local thyroid clock, which was still ticking after hypophysectomy.

## Declaration of interest

The authors declare that the research was conducted with no commercial or financial relationship that could be interpreted as a potential conflict of interest.

## Funding

This work was supported by the Danish Biotechnology Center for Cellular Communication.

## Author contribution statement

J F designed the experiments. B G performed the *in vivo* experiments and real-time quantitative PCR. J H performed immunohistochemistry and made the figure. H L J performed the statistics and made the remaining figures. J F wrote the manuscript and all authors reviewed and edited the manuscript.
